# Modeling congenital kidney diseases in *Xenopus laevis*

**DOI:** 10.1242/dmm.038604

**Published:** 2019-04-09

**Authors:** Alexandria T. M. Blackburn, Rachel K. Miller

**Affiliations:** 1 Pediatric Research Center, Department of Pediatrics, McGovern Medical School, The University of Texas Health Science Center, Houston, TX 77030, USA; 2 The University of Texas MD Anderson Cancer Center UTHealth Graduate School of Biomedical Sciences, Program in Genetics and Epigenetics, Houston, TX 77030, USA; 3 The University of Texas MD Anderson Cancer Center UTHealth Graduate School of Biomedical Sciences, Program in Biochemistry and Cell Biology Houston, Houston, TX 77030, USA; 4 Department of Genetics, The University of Texas MD Anderson Cancer Center, Houston, TX 77030, USA

**Keywords:** *Xenopus*, Nephron, CAKUT, Nephronophthisis, PKD, CRISPR, Kidney

## Abstract

Congenital anomalies of the kidney and urinary tract (CAKUT) occur in ∼1/500 live births and are a leading cause of pediatric kidney failure. With an average wait time of 3-5 years for a kidney transplant, the need is high for the development of new strategies aimed at reducing the incidence of CAKUT and preserving renal function. Next-generation sequencing has uncovered a significant number of putative causal genes, but a simple and efficient model system to examine the function of CAKUT genes is needed. *Xenopus laevis* (frog) embryos are well-suited to model congenital kidney diseases and to explore the mechanisms that cause these developmental defects. *Xenopus* has many advantages for studying the kidney: the embryos develop externally and are easily manipulated with microinjections, they have a functional kidney in ∼2 days, and 79% of identified human disease genes have a verified ortholog in *Xenopus*. This facilitates high-throughput screening of candidate CAKUT-causing genes. In this Review, we present the similarities between *Xenopus* and mammalian kidneys, highlight studies of CAKUT-causing genes in *Xenopus* and describe how common kidney diseases have been modeled successfully in this model organism*.* Additionally, we discuss several molecular pathways associated with kidney disease that have been studied in *Xenopus* and demonstrate why it is a useful model for studying human kidney diseases.

## Abstract (precis)

**Summary:** Understanding how congenital kidney diseases arise is imperative to their treatment. Using *Xenopus* as a model will aid in elucidating kidney development and congenital kidney diseases.

## Modeling CAKUT in *Xenopus*

**Fig. 1.**
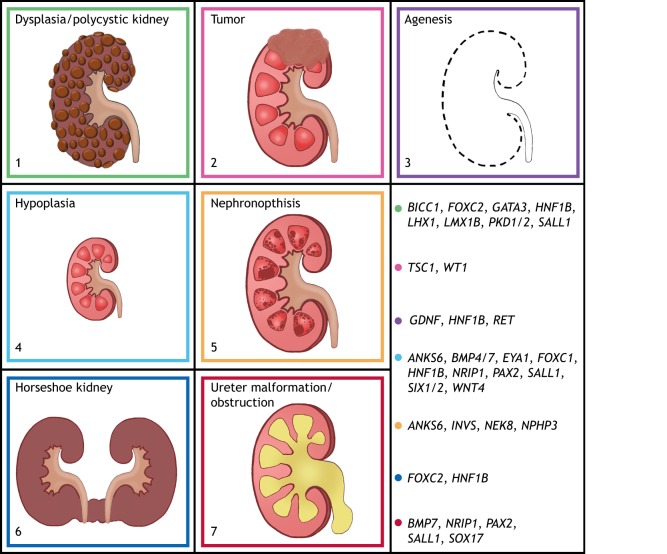
**Common malformations of the kidney found in CAKUT.** Renal malformations resulting from inherited kidney diseases are depicted in colored boxes. The colors correspond to the color coding for genes whose loss results in the given phenotype. (1) Green represents renal cysts that are large and cover the majority of the kidney, as seen in renal dysplasia, multicystic dysplastic kidney (MCDK) and autosomal-dominant polycystic kidney disease (ADPKD). (2) Magenta represents a tumor, as seen in tuberous sclerosis and Wilms tumor. (3) Purple represents kidney agenesis (Box 1). (4) Teal represents renal hypoplasia, which is one of the most common CAKUT phenotypes. (5) Orange represents nephronophthisis, with maroon spots depicting corticomedullary cysts, which are generally small. (6) Blue represents the horseshoe kidney, where both kidneys are fused together. (7) Red represents ureter malformations and blockages, which result in urine backflow into the kidney (shown in yellow in the schematic). Genes listed in the key have been studied or are expressed in the *Xenopus* kidney. Numbers found in this figure in the bottom left corner that correspond to the aforementioned phenotypes can also be found in Table 1 under ‘Renal phenotype’.

Congenital anomalies of the kidney and urinary tract (CAKUT; see Box 1 for a glossary of terms) are a leading cause of pediatric kidney failure, accounting for 40-50% of pediatric chronic kidney disease (CKD; Box 1) worldwide (Vivante et al., 2014). CAKUT encompasses a wide range of structural malformations resulting from morphogenetic defects, including Wilms tumor and renal hypodysplasia (Box 1; Fig. 1) (Yosypiv, 2012). With an average 3- to 5-year wait time for a deceased donor kidney transplant [United Network for Organ Sharing (https://www.kidney.org/atoz/content/transplant-waitlist), accessed December 10, 2018], the need to find alternative treatments that preserve renal function is essential. Monogenic disease with strong genetic causality only accounts for 12% of CAKUT cases (Vivante and Hildebrandt, 2016), and polygenic causes are speculated to occur but are largely unknown (Sanna-Cherchi et al., 2018). Next-generation sequencing has helped to uncover novel causative genes of CAKUT, but a high-throughput strategy to test their function in the kidney is needed. To understand how CAKUT arises, it is crucial to understand how the kidneys and urinary tract develop to uncover the genetic mechanisms that coordinate these events. Mice and zebrafish have been the predominant models used in kidney research, while recent advances have made kidney organoids (Box 1) useful for nephrotoxicity screening as well as for modeling kidney diseases (Hurtado del Pozo et al., 2018). However, *Xenopus laevis* frogs (Box 1; hereafter referred to simply as *Xenopus*) possess many qualities that make them an effective *in vivo* model to study congenital kidney diseases.

**Box 1. Glossary**

**Ciliopathy:** a genetic disorder caused by abnormal formation or function of cilia (a component of almost all cells). Disruption of cilia leads to a recognizable list of features, including retinal degeneration, cardiac defects, mental retardation and kidney disease.

**Epigenetic signature:** a set of epigenetic marks, such as methylation, found on specific genes that are associated with the phenomenon being observed, such as a disease state.

**Excretion assay:** fluorescent dyes, such as rhodamine, can be injected into the *Xenopus* coelomic cavity and filtered by the kidneys. These dyes are then secreted into the urine, which allows for a simple visual readout of kidney function.

**Expressivity:** measures the extent to which a given genotype manifests (is expressed) at the phenotypic level. It accounts for different degrees of phenotypic expression in different individuals, which may be due to environmental factors or the allelic constitution of the rest of the genome.

**Fate mapping:** determines what types of cells, tissues and organs are derived from specific embryonic cells. Classical fate maps inject a lineage tracer such as a fluorescent dextran into a specific cell, which then allows all of its descendants to retain the fluorescence and therefore be mapped.

**GAL4-UAS:** involves the development of two lines: the GAL4 line, which expresses the transcription factor GAL4 in a subset of the animal's tissues; and the UAS line, in which UAS is normally expressed upstream of fluorescent proteins and acts as a reporter. GAL4 specifically binds to UAS promoter elements, thus activating expression of the downstream target sequence.

**Genome-wide association study (GWAS):** an observational study of the entire genome and its set of genetic variants in different individuals to see whether any variant may be associated with a specific trait. These studies generally focus on single-nucleotide variants and associations that may lead to a predisposition of various diseases.

**Glomerulopathy:** a disease affecting the glomeruli of the nephron that causes the kidneys to malfunction. Features include high levels of protein and sometimes blood in the urine, and swelling in many areas of the body. Loss of glomerular filtration leads to end-stage renal disease (ESRD) in about half of the individuals within 10 years of their diagnosis.

**Heat-shock inducible:** a heat shock promoter, normally the *Hsp70* promoter, is used to regulate transgene expression when the ambient temperature is briefly increased. This heat shock releases a factor that then allows it to bind to elements of the promoter, thus activating transcription. This technique has been used in aquatic animals such as zebrafish and *Xenopus*, as well as in *Caenorhabditis* *elegans* and *Drosophila*.

**Intermediate mesoderm:** part of the mesoderm germ layer that is located between the paraxial and lateral plate mesoderm. The intermediate mesoderm gives rise to the reproductive and urogenital systems, including the kidney.

**Kidney organoids:** result from kidney organogenesis in a dish (*in vitro*) by inducing human pluripotent stem cells to a kidney fate. The organoids contain cell clusters that express markers of various regions of the kidney, such as podocytes, proximal tubules, loops of Henle and the distal tubule. Additionally, *Xenopus* explants of early embryos can be induced to form kidney organoids in culture.

**Leapfrogging:** transplanting the germline of a *Xenopus* embryo that has been mutagenized (such as with CRISPR/Cas9) into a wild-type host that had its wild-type germline removed. This results in the efficient transmission of mutant alleles to F_1_ offspring and overcomes the embryonic lethality of various gene knockouts in the F_0_ embryos.

**Meckel-Gruber syndrome:** a rare autosomal recessive ciliopathy characterized by renal cystic dysplasia, polydactyly and central nervous system malformations. Most individuals with Meckel-Gruber syndrome die before or shortly after birth.

**Mesonephros:** Greek for ‘middle kidney’; the main excretory organ of aquatic vertebrates and a temporary kidney in reptiles, birds and mammals. It develops posterior to and replaces the pronephros. In humans, the mesonephros functions between the sixth and tenth weeks of embryological life.

**Metanephric mesenchyme** **(MM):** all of the cells present in mature nephrons arise from the MM. During kidney development, cells of the MM condense around the ureteric bud to form what's known as the cap mesenchyme, which goes on to form the renal vesicles, then the comma-shaped bodies and so forth.

**Metanephros:** the third stage of kidney development, which corresponds to the mature and functional kidney in reptiles, birds and mammals. In humans, the metanephros develops by the tenth week of embryological life, replacing the mesonephros.

**Morpholino:** antisense oligonucleotide that binds to complementary RNA to knock down gene expression. Its molecular structure contains DNA bases that are attached to a backbone of methylmorpholine rings linked to non-ionic phosphorodiamidate linkages instead of anionic phosphodiester ones, which make them highly stable.

**Nail-patella syndrome:** an autosomal-dominant disease marked by poorly developed nails, kneecaps (patellae), elbows and pelvis, as well as kidney disease. Nail-patella syndrome is caused by mutations in *LMX1B*.

**Nephric primordia:** the primordia is the simplest set of cells that are specified to become the kidney and the earliest recognizable stage of kidney development; also known as pronephric anlage.

**Nephron:** the structural and functional unit of the kidney that filters body waste and excess fluid. The nephron is composed of a glomerulus to filter blood; proximal tubules, a loop of Henle and distal tubules for reabsorbing water and various nutrients; and a collecting duct that resorbs water and carries urine from the kidneys to the bladder.

**Nephronophthisis (NPHP):** an autosomal-recessive ciliopathy that generally occurs as an isolated kidney disease, although ∼15% of NPHP patients also present with extrarenal symptoms. Features include small cysts in the kidney medulla and kidney fibrosis, with three clinical forms classified by the onset of ESRD: infantile, juvenile and adolescent.

**Nephrostomes:** multi-ciliated cells that funnel blood filtrate from the coelomic cavity and into the tubules of the *Xenopus* kidney.

**Neurula:** the embryonic stage of development in which neurulation, the transformation of the neural plate into the neural tube, occurs. This process begins when the notochord induces the formation of the central nervous system by forming the neural plate.

**Oocyte:** an immature female egg that matures within a follicle in the outer layer of the ovaries. *Xenopus* oocytes have been commonly used to study ion transport and channel physiology because of their large diameter (1 mm), as well as making cell-free extracts to study cell and molecular biology.

**Polycystin signaling:** biochemical interactions between proteins that lead to cyst formation in the kidney, including HNF1B, TSC2 and BICC1, and the *PKD1* and *PKD2* gene products, polycystin 1 and polycystin 2. Cyst formation may occur due to their downstream signaling pathways or due to the direct interactions between these proteins.

**Pronephros:** the first stage of kidney development that is functional in aquatic vertebrates such as *Xenopus*, but nonfunctional in reptiles, birds and mammals. In humans, the pronephros is a vestigial structure that disappears completely by the fourth week of embryonic life. Despite this transient appearance in mammals, the pronephros is essential for the development of the adult kidneys.

**Renal vesicles:** the primordial structure of the nephron that is the first polarized epithelial derivative of the metanephric mesenchyme. Renal vesicles sequentially evolve into the comma-shaped body, followed by the S-shaped body and finally the nephron.

**Retinoic acid signaling:** a concentration-dependent signaling pathway important for embryo patterning and development. Retinoic acid signaling is essential for kidney development and regulates embryonic kidney patterning.

**Septins:** a group of GTP-binding proteins that form complexes including filaments and rings. Septins are a unique cytoskeletal component that have been implicated in the localization of proteins at the cell membrane where cilia are found.

**S-shaped bodies:** the epithelial precursors for the nephron segments (glomerulus, proximal tubule and distal tubule). The segments are oriented along the proximal-distal axis with the glomerulus being the most proximal.

**Tet-On:** an inducible gene expression system that requires two lines, one carrying the gene of interest under the control of a tetracycline-inducible promoter (fusion of CMV minimal promoter and seven copies of the *tetO* sequence) and the other carrying the transcriptional activator, rtTA. In the absence of rtTA, the CMV minimal promoter cannot drive the expression of the transgene. However, when rtTA binds to the *tetO* sequences, RNA polymerase can be recruited to the CMV minimum promoter to begin transcription.

**Tubulopathy:** a disease that affects the tubules of the nephrons in the kidney. It can arise from mutations in ion channel genes such as *CLCNKB*. Tubular dysfunction can cause profound electrolyte and volume disturbance.

**Wilms tumor:** a type of childhood cancer in the kidneys that most often affects children aged 3 to 4 years. Most Wilms tumors only affect one kidney and the chance of curing children with these tumors is very high.

**Wnt pathway:** a group of signaling pathways that are essential for embryonic development and are highly conserved in animals, from fruit flies to humans. There are three main branches of the Wnt pathway: the canonical Wnt pathway involving β-catenin, the non-canonical planar cell polarity pathway, and the non-canonical Wnt/calcium pathway. All three pathways are activated when a Wnt ligand binds to a Frizzled receptor and transduces the signal to the Dishevelled protein. Both canonical and non-canonical planar cell polarity pathways are important for kidney development, with potential involvement of the calcium pathway (Halt and Vainio, 2014).

***Xenopus laevis*:** an aquatic species of African clawed frogs that have been used as a model organism to study embryonic development, cell and molecular biology, as well as in large-scale genetic screens. *Xenopus laevis* is an established model of nephron development.

**Table 1.**
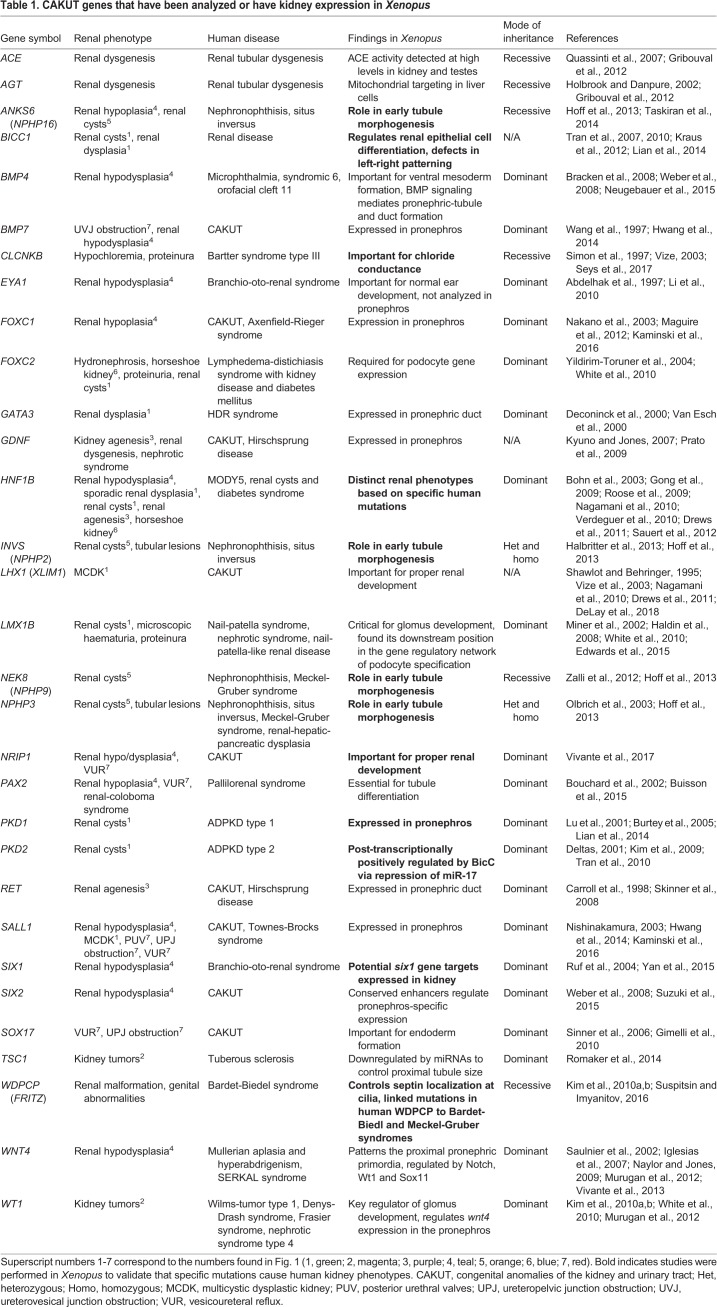
**CAKUT genes that have been analyzed or have kidney expression in *Xenopus***

**Fig. 2.**
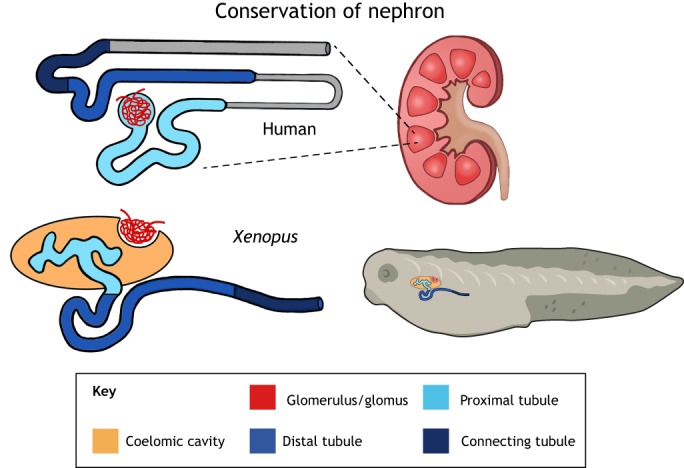
***Xenopus* pronephric and human metanephric nephrons share conserved tubule segmentation patterns based on gene expression data.** The schematic represents nephron segments, designated by different colors, in the mammalian metanephric nephron (top) and the *Xenopus* pronephric nephron (bottom). The glomus/glomerulus filters blood across capillary walls into the proximal tubule, which filters various wastes out of the body through the remaining distal and connecting tubules. There are noted differences between the glomus and glomerulus in that the glomus deposits blood filtrate into the coelomic cavity. Additionally, *Xenopus* does not have a loop of Henle (grayed out in human schematic) or a true collecting duct (grayed out in human schematic), but instead has a region analogous to the connecting tubule closest to the distal tubule of the mammalian metanephric nephron (Raciti et al., 2008).

*Xenopus* share a relatively close evolutionary history with mammals because frogs are tetrapods. Thus, this model has the advantage of rapid development, like zebrafish, but evolutionarily it lies closer to mammals. Additionally, *Xenopus* and human genomes have long stretches of gene collinearity, and 79% of identified human disease genes have a verified ortholog in *Xenopus* (Hellsten et al., 2010). The embryonic kidney of *Xenopus* has many characteristic features of a mature mammalian kidney (Fig. 2) (Zhou and Vize, 2004; Raciti et al., 2008). Thus, many genes and processes necessary for *Xenopus* kidney development are also important in mammalian kidney development. Furthermore, a limited number of kidney-disease-causing genes have been analyzed in *Xenopus* or are expressed in its embryonic kidney, introducing potential paths for future kidney research (Fig. 1 and Table 1). Although few in number, the *Xenopus* genes studied have been shown to function in kidney development, as their mammalian orthologs do in mammalian kidneys, and their disrupted expression results in similar nephron (Box 1) phenotypes to those seen in humans. Additionally, *Xenopus* possesses unique qualities that other *in vivo* model systems lack.

With a simple hormone injection, *Xenopus* produces large clutch sizes with hundreds of embryos that develop externally. Their kidneys can be easily visualized and imaged through their transparent epidermis, and they develop a fully functional kidney in ∼56 hours post-fertilization (hpf) (Vize et al., 2003). Additionally, cleavage-stage *Xenopus* embryos have been fate-mapped (Box 1), which allows for the tracking of specific blastomeres (Box 1) as they develop from an early-stage embryo into a differentiated body plan (Dale and Slack, 1987; Moody, 1987). This facilitates unilateral tissue-targeted injections that are specific to organs such as the kidney (DeLay et al., 2016) and permits using the uninjected side as an internal control. In addition, researchers have developed assays to study kidney function in *Xenopus* (Zhou and Vize, 2004; Tran et al., 2010).

*Xenopus* oocytes (Box 1) and early embryos can easily be injected with DNA, mRNA, protein and/or morpholinos (Box 1) to overexpress or knock down proteins. A common assay in *Xenopus* involves disrupting genes/proteins in both kidneys by injecting morpholinos, or CRISPR sgRNAs and the Cas9 protein, in both ventral cells of four-cell embryos. If the gene/protein is important for kidney formation, its loss will result in edema, characterized by swelling in the chest cavity due to fluid retention. This technique allows for disruption of the kidney while avoiding the heart and liver, two other common causes of edema, generating tissue-targeted knockdown or knockout embryos. Upon edema formation, researchers can then use an excretion assay (Box 1) that allows visualization of the passage of fluorescent molecules through the kidney and out through the cloaca, which can show whether the kidneys are still functioning (Zhou and Vize, 2004). Although not directly comparable to the Cre-*loxP* systems used in mouse studies, this edema assay, which is unique to *Xenopus*, allows for some evaluation of tissue specificity. Knockdown of Pkd2, a protein involved in autosomal-dominant polycystic kidney disease (ADPKD; Box 1), in *Xenopus* embryonic kidneys results in edema (Tran et al., 2010). Similar edema phenotypes occur due to the loss of other proteins implicated in CAKUT, such as Pax2 and Pax8 (Buisson et al., 2015). These established techniques demonstrate that *Xenopus* possesses distinctive qualities suitable for disease modeling, and new technologies continue to improve the experimental opportunities in this model system.

Genetic screens of the allotetraploid (Box 1) *Xenopus* are becoming more feasible with the development of the CRISPR/Cas9 system (Aslan et al., 2017), and the ability to target CRISPR-mediated genetic manipulation to the kidney is unique to *Xenopus* (DeLay et al., 2018). These advantages make *Xenopus* a valuable model for studying the kidney, can aid in our understanding of the mechanisms through which the kidney develops, and can help identify new genes important for renal function. This Review will highlight the *Xenopus* genes and pathways that are associated with human kidney disease, demonstrate the similarity in phenotypes upon gene or protein disruption, and establish *Xenopus* as a relevant model for CAKUT.

## Kidney development

The kidney is an excretory organ that filters blood. It functions by reabsorbing nutrients and excreting waste and excess fluid in the form of urine. In humans, nephrogenesis starts at around 5 weeks of gestation and completes between weeks 34 and 36 (Black et al., 2012). Environmental factors, such as vitamin A deficiency or exposure to teratogenic substances such as angiotensin-converting enzyme (ACE) inhibitors, among others, can affect proper kidney development (Rosselot et al., 2010; Al-Maawali et al., 2012). Retinoic acid is derived from vitamin A and retinoic acid signaling (Box 1) is important for embryonic kidney patterning (Rosselot et al., 2010). Additionally, taking teratogenic ACE inhibitors during pregnancy can lead to CAKUT, as the kidney also plays an essential role in controlling blood pressure (Al-Maawali et al., 2012). The kidney is a unique organ in that it develops from successive forms that replace the previous structure (Davidson, 2008). These forms are the pronephros, mesonephros and metanephros (Box 1). The organizational complexity increases as each sequential form of the kidney is superseded by the next (Davidson, 2008). In mammals, both the pro- and mesonephros are replaced by the metanephros, which persists to function as the adult kidney (Little and McMahon, 2012). In contrast, the onset of metamorphosis in *Xenopus* marks the replacement of the pronephros with the mesonephros, which is the adult kidney of amphibians. It is important to note that, although the pronephros is non-functional in mammals, it is required for the subsequent formation of the mesonephros and metanephros.

Although the arrangement of kidneys differ, the structural and functional unit of the kidney, the nephron, remains the same. An adult human kidney contains approximately 1 million nephrons (Bertram et al., 2011), whereas an adult mouse kidney has over 10,000 (Short et al., 2014). Mammalian nephrons develop asynchronously, making them challenging to study (Sorokin et al., 1992). In contrast, *Xenopus* embryos contain only one functional nephron on either side of their body, which serves as the embryonic kidney. Therefore, the fully functional *Xenopus* pronephric nephron serves as a simplified model for mammalian meso- and metanephric nephron development (Fig. 2).

## Conservation of the nephron in *Xenopus* and mammals

The pronephros forms from the intermediate mesoderm (Box 1) in both mammals and amphibians*.* In *Xenopus*, the nephric primordia (Box 1) is induced by signals from the surrounding tissues and forms during neurula (Box 1) stages 12-15 (∼16-20 hpf), and later generates the tubules of the pronephros (Fox, 1963). Pronephros specification in *Xenopus* occurs through the interactions between the transcription factors Osr1, Osr2, Pax8, Lhx1 and Hnf1B, among others (Vize et al., 2003; Tena et al., 2007; Drews et al., 2011; Boualia et al., 2013). Morphogenesis of the pronephros begins at the early-tailbud stage 21 (∼23 hpf) (http://www.xenbase.org/anatomy/alldev.do). In mice, the *Osr1* gene is essential for the formation of renal structures and can be found in the intermediate mesoderm, while *Osr2* is found later on in the mesonephros and is not essential (Tena et al., 2007). Consistent with *Xenopus* (Buisson et al., 2015; DeLay et al., 2018), mice that lack Lhx1 or both Pax2 and Pax8 have severe kidney defects (Pax2 and Pax8 seem to have redundant functions in mice) (Shawlot and Behringer, 1995; Bouchard et al., 2002). Finally, Hnf1B is expressed in the ureteric bud as well as in comma- and S-shaped bodies (Box 1), and mutations in this gene lead to kidney cyst formation in mice (Gong et al., 2009). In addition, Wnt (wingless/integrated; Box 1), FGF (fibroblast growth factor), BMP (bone morphogenetic protein) and GDNF (glial-cell-line-derived neurotrophic factor) signaling pathways are involved in both *Xenopus* and mammalian kidney development (Dressler, 2006; Costantini and Kopan, 2010).

Analogous to the mammalian nephron, the amphibian pronephros is segmented along its proximal-distal axis (Møbjerg et al., 2000). Mammals have a glomerulus to filter blood, proximal tubules, a loop of Henle and distal tubules for reabsorbing water and various nutrients, and a collecting duct that reabsorbs water and carries urine from the kidneys to the bladder (Little and McMahon, 2012). Similarly, *Xenopus* embryos have a glomus for filtering blood, proximal and distal tubules for reabsorbing nutrients and a connecting tubule, which opens out to the cloaca (Fig. 2). In addition, at the tips of the proximal tubules, the amphibian also has multi-ciliated cells called nephrostomes (Box 1), which cause fluid influx from the coelomic cavity (Box 1) into the proximal tubules (Wessely and Tran, 2011).

Each segment of the nephron has a specific function, which is reflected by distinct cell morphologies (Møbjerg et al., 2000) and gene expression signatures (Zhou and Vize, 2004; Raciti et al., 2008). Strikingly similar to mammals, the proximal tubules in *Xenopus* are responsible for reabsorbing ions, water, glucose and amino acids (Eid et al., 2002; Zhou and Vize, 2004, 2005; Christensen et al., 2008; Raciti et al., 2008). The most distal part of the *Xenopus* proximal tubule is analogous to the mammalian proximal straight tubule (Raciti et al., 2008). Because *Xenopus* is an aquatic freshwater frog, it is unlikely that the amphibian pronephros requires a true loop of Henle for concentrating urine. Within the literature, the *Xenopus* intermediate tubules have been defined using markers that are co-expressed in the proximal and distal tubules of the mouse kidney (Reggiani et al., 2007; Raciti et al., 2008). Thus, assessment of a marker that is exclusive to this region is necessary to confirm or refute the existence of this structure. The *Xenopus* distal tubule is analogous to the thick ascending limb of the loop of Henle and the distal convoluted tubule in mammals (Raciti et al., 2008); it functions to transport ammonium and reabsorb magnesium ions, and is important for urine acidification (Vize, 2003; Zhou and Vize, 2004, 2005; Raciti et al., 2008). The pronephric connecting tubule of *Xenopus* connects the distal tubules to the cloaca to excrete urine and is only analogous to mammals in the region neighboring the distal tubule (Raciti et al., 2008). The pronephros does not have a collecting duct to further concentrate urine or connect multiple nephrons to a secondary structure, as is necessary for the mammalian metanephros.

**Fig. 3.**
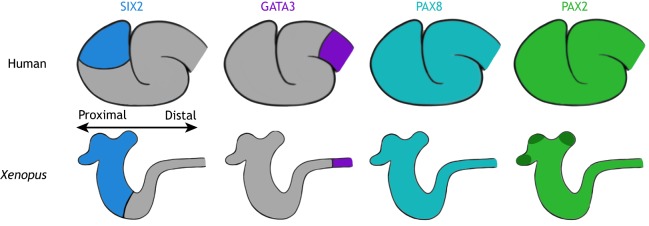
**Expression patterns are conserved between the human S-shaped body and the early *Xenopus* pronephros during development*.*** Four different expression patterns (shown in different colors) of fundamental kidney proteins/mRNA were chosen to demonstrate the similarities between the developing human and *Xenopus* nephron*.* Schematics indicate where immunostaining of S-shaped body nephrons of week-16 to -17 human fetal kidneys is present (top) (Lindström et al., 2018), as well as *in situ* expression patterns of stage-33 *Xenopus* nephrons (bottom) (Xenbase.org). Schematics are positioned so that the proximal and distal regions of the human and *Xenopus* nephron expression patterns can be easily compared. The *Xenopus* kidney stage was chosen to match the approximate developmental time point of human S-shaped body nephrons, as both are representative of recently epithelialized nephrons. Note that *pax2* is slightly more enriched at the nephrostomes in *Xenopus* (dark green).

Recent research has shown that human and mouse nephron protein-expression signatures in the renal vesicles (Box 1) and S-shaped bodies are very similar (Lindström et al., 2018). Not surprisingly, *Xenopus* shares similar gene expression signatures to humans during early pronephric development in the kidney (Fig. 3). Although Fig. 3 only illustrates four gene expression signatures, it is important to note that, of the 29 expression signatures identified in the human embryonic kidney (Lindström et al., 2018), 18 can also be found in the *Xenopus* kidney, including *mafb*, *slc3a1*, *pou3f3* and *foxc2* (xenbase.org). Additionally, a comparison of gross expression signatures in the kidney demonstrated similarities between mouse and *Xenopus* in both overlapping transcription factors such as *Pax8* and additional ones such as *Foxc1* and *Sall1* (Kaminski et al., 2016)*.* Furthermore, some of the proteins analyzed in humans have not been studied in *Xenopus*, potentially leading to an underestimate of the similarities. This overall conservation on the molecular, genetic, structural and functional levels supports the use of the *Xenopus* pronephros as an appropriate model for the study of CAKUT.

## Delineating molecular pathways involved in kidney development using *Xenopus*

*Xenopus* has been historically used to elucidate molecular mechanisms and signaling cascades involved in early developmental processes (Beck and Slack, 2001). More recently, *Xenopus* has played an important role in identifying the function of genes that are involved in CAKUT. The Wnt pathway has been heavily studied in *Xenopus* due to this pathway's significant contribution to various developmental processes. The canonical Wnt/β-catenin pathway plays a role in kidney development in both mice (Carroll et al., 2005; Iglesias et al., 2007) and *Xenopus* (Saulnier et al., 2002; Lyons et al., 2009). WNT4 is a well-known ligand that activates canonical Wnt signaling and is important for induction and mesenchyme-to-epithelial transition of the metanephric mesenchyme (Box 1) (Murugan et al., 2012). In humans, *WNT4* loss-of-function mutations have been associated with renal hypodysplasia, a condition characterized by dysplastic kidneys with a reduced number of nephrons (Vivante et al., 2013). Studies in *Xenopus* demonstrate that Wnt4 protein controls the medio-lateral patterning of the pronephros (Naylor and Jones, 2009). Additionally, knockdown of Wnt4 protein in *Xenopus* results in complete loss of kidney tubules (Saulnier et al., 2002).

In addition to Wnt ligands, other proteins have been found to regulate Wnt signaling. In humans, Wilms tumor 1 (*WT1*) is a known regulator of canonical Wnt signaling. Mutations that affect *WT1* result in nephroblastoma, more commonly known as Wilms tumor (Kim et al., 2010a,b). Knockdown of Wt1 protein expression in *Xenopus* embryos was shown to reduce Wnt4 expression in the prospective pronephros (Murugan et al., 2012). Mutations in the RNA-binding protein BICC1, a negative Wnt regulator, result in a cystic kidney phenotype in mice and renal anomalies in patients (Kraus et al., 2012). Prior studies in *Xenopus* indicate that Bicc1 inhibits the microRNA miR-17, preventing miR-17 from destabilizing the *pkd2* transcript (Tran et al., 2010). This was demonstrated by a rescue of the mutant kidney phenotype when both miR-17 and Bicc1 were simultaneously knocked down in tadpoles (Tran et al., 2010). Interestingly, loss of the *Pkd1* gene product, polycystin-1, downregulates BICC1 expression in mouse kidneys and mouse cell lines (Lian et al., 2014). The connection between BICC1 downregulation and polycystin-1 loss in mice and the stabilization of *pkd2* mRNA by Bicc1 in *Xenopus* suggests that disruption of *BICC1* may induce cystic phenotypes through polycystin signaling (Box 1). Similarly, targeted deletion of *Hnf1b*, a key transcription factor involved in kidney development, decreases *Bicc1* and *Pkd2* mRNA expression in the mouse kidney (Verdeguer et al., 2010). HNF1B in the mouse (Gresh et al., 2004; Verdeguer et al., 2010) and BICC1 in mice and *Xenopus* (Tran et al., 2010) regulate the *Pkd2* transcript, and mutations in either gene can lead to kidney cysts in mouse models (Gong et al., 2009; Tran et al., 2010). Thus, it is likely that these genes act through similar pathways. *Xenopus* would be an ideal model for studying the components of this pathway because molecular strategies to assess them have already been established (Tran et al., 2007, 2010).

Another common congenital kidney disease whose pathway has been elucidated using *Xenopus* is nephronophthisis (NPHP; Box 1). NPHP is a ciliopathy (Box 1), resulting in either abnormal formation or function of cilia (Box 1). NPHP is a rare birth defect, but it is the most common cause of kidney failure, or end-stage renal disease (ESRD; Box 1), in the first three decades of life (Wolf and Hildebrandt, 2011). Studies in *Xenopus* linked several genes with NPHP or clarified components of the mechanism that causes the disease. First, targeted kidney knockdown of the Wnt signaling inhibitor Invs (inversin; also known as NPHP2) demonstrated that Invs is important for morphogenetic cell movements during tubule elongation (Lienkamp et al., 2010). This knockdown led to impaired ventral proximal pronephros extension and distal tubule differentiation (Lienkamp et al., 2010). Later, it was discovered that several genes that cause NPHP form a distinct complex that contributes to kidney development. In *Xenopus*, Anks6 was identified as an NPHP family member that assembles a protein complex of the NPHP-associated proteins Nek8, Invs and Nphp3 to regulate kidney development (Hoff et al., 2013). This study showed that Anks6 localizes to the base of the cilium and that knockdown of its protein expression results in kidney anomalies (Hoff et al., 2013). The study also linked *ANKS6* mutations in patients with an NPHP-like clinical syndrome (Hoff et al., 2013). Additionally, knockdown of either Anks6 or Nphp3 protein in *Xenopus* embryos results in edema, which suggests that they may be important for kidney function (Hoff et al., 2013). Likewise, edema has been shown to occur due to depletion of Invs protein (Lienkamp et al., 2010).

Although *Xenopus* has helped uncover some of the causative genes for NPHP and their role in kidney development and function, only ∼40% of patients have a mutation in one of the 20 known NPHP-related genes (Stokman et al., 2016; Srivastava et al., 2018). A recent genome-wide association study (GWAS; Box 1) has uncovered epigenetic signatures of CKD (Box 1), including *NPHP4* (Wing et al., 2014). Some NPHP patients may suffer from epigenetic alterations of NPHP genes rather than a mutation in the gene itself. Future research using *Xenopus* may lead to the identification of new NPHP-causing genes and may uncover further epigenetic regulation of known genes as they have been used to study epigenetics.

A notable CAKUT-causing gene encodes the transcription factor LMX1B, mutations in which can lead to nail-patella syndrome (NPS; Box 1), a rare cause of autosomal-dominant ESRD (Edwards et al., 2015). Additionally, mutations in *LMX1B* can result in glomerulopathies (Box 1). Mouse studies suggest that *Lmx1b* is necessary for the maintenance of podocytes, which are highly specialized cells that wrap around capillaries of the glomerulus and restrict the passing of macromolecules into the kidney (Miner et al., 2002). In *Xenopus*, *lmx1b* is expressed in the glomus, which is analogous to the mammalian glomerulus, and is also known to have a role in podocyte specification (Haldin et al., 2008; White et al., 2010). Morpholino knockdown of Lmx1b protein in *Xenopus* showed that the glomus was reduced in size and the development of the proximal tubules was limited (Haldin et al., 2008). Taken together, these studies demonstrate the usefulness of *Xenopus* in uncovering the role of proteins important for kidney development and function. Additionally, they show that *Xenopus* is capable of recapitulating specific kidney disease phenotypes found in humans.

## Using *Xenopus* to examine human genetic variants

In human studies, missense mutations are often analyzed via software programs such as PredictSNP (Bendl et al., 2014) and Meta-SNP (Capriotti et al., 2013). These programs predict the functional relevance of an amino acid change and suggest whether they are likely to be pathogenic. One drawback of this method is the surprising data from the 1000 Genomes Project indicating that people with predicted pathogenic variants do not suffer from the expected disease (Xue et al., 2012). *Xenopus* can be used to verify the pathogenicity and expressivity (Box 1) of potential disease-causing genetic variants *in vivo*.

Work in mouse models found that CLCNKB is important for concentrating urine (Matsumura et al., 1999). In humans, *CLCNKB* mutations cause a salt-losing tubulopathy (Box 1) known as Bartter syndrome type III (Simon et al., 1997). The first genotype-phenotype correlation for Bartter syndrome type III was validated by functional analysis of eight missense and two nonsense mutations in *Xenopus*. By performing voltage-clamp experiments in *Xenopus* oocytes, it was found that nine of the ten mutations significantly decreased normal conductance (Seys et al., 2017), indicating altered salt homeostasis. This work verified that the reported human genetic mutations were, in fact, Bartter syndrome type III-causing mutations (Seys et al., 2017). A similar study demonstrated that dysregulation of NRIP1-dependent retinoic acid signaling in both *Xenopus* and mouse disrupted kidney formation (Vivante et al., 2017). In *Xenopus*, knockdown of Nrip1 protein causes kidney anomalies that can be rescued with wild-type human *NRIP1* mRNA but not with the truncated *NRIP1* mRNA identified in affected individuals (Vivante et al., 2017). *Xenopus* can also serve to assess the distinct effects of different mutations in a single gene.

*HNF1B* mutations have been shown to manifest as distinct renal diseases in humans (Bohn et al., 2003). An attempt to model these differences in *Xenopus* was successfully executed using different methods in two separate studies. In one study, researchers generated transgenic *Xenopus* lines expressing two different human *HNF1B* mutants using a heat-shock-inducible (Box 1) Cre-*loxP* system (Sauert et al., 2012). One transgenic line expressed an insertion mutation, while the other line expressed a deletion. The deletion led to reduced pronephric development, while the insertion enlarged the pronephros, with both phenotypes primarily affecting the proximal tubules (Sauert et al., 2012). In another study, Bohn and colleagues compared nine different human *HNF1B* mutations, including indels, missense and nonsense mutations, by injecting mutant mRNA. The mutations led to distinct renal disease phenotypes in *Xenopus*, as they do in humans (Bohn et al., 2003). In *Xenopus*, six of the mutants resulted in an enlargement of the pronephric structures, while the other three mutations led to a reduction or loss of the tubules and the anterior part of the duct (Bohn et al., 2003). The range of kidney phenotypes seen in *Xenopus* recapitulate the kidney phenotypes observed in humans. This potentially allows for a correlation between an observed patient phenotype with a specific mutation in the *HNF1B* gene. Studies like these are an efficient way to identify pathogenic genetic variants and can represent the variation of expressivity seen in patients.

## Early embryonic lethality

In *Xenopus*, many genes that are involved in kidney development have essential functions in other tissues. To bypass embryonic lethality due to the loss of these essential proteins, researchers have developed techniques that are not possible in other organisms. For example, tissue-targeted microinjection has been used to study ADPKD, the most common inherited human renal disease. ADPKD is caused by mutations in either *PKD1* or *PKD2*, with *PKD2* mutations causing a less severe form of ADPKD with a later onset than *PKD1* mutations (Deltas, 2001). Homozygous mutations or deletions of either *Pkd1* or *Pkd2* in mouse models are embryonic lethal (Lu et al., 2001; Kim et al., 2009). This limitation can be resolved by tissue-targeted microinjections in *Xenopus* as described earlier, as both *pkd1* (Burtey et al., 2005) and *pkd2* (Tran et al., 2010) are expressed in the *Xenopus* embryonic kidney. Using this technique, Tran and colleagues elucidated some aspects of *pkd2* post-transcriptional regulation (Tran et al., 2010). Although a variety of mouse models have since been developed to bypass the embryonic lethality of a complete knockout mouse, there is still a need for ADPKD models that more closely resemble the human disease. *Xenopus* has a lifespan that permits an extended survival that would add additional information of how ADPKD progresses over time. Additionally, elucidating the mechanism of the disease using *Xenopus* may be more cost and time effective.

Inducible systems have also been successfully used in *Xenopus* embryos, which allows for temporal or spatial control of gene expression. Four inducible systems were developed in *Xenopus*: the GAL4-UAS (Box 1) (Denayer et al., 2006), Tet-On (Box 1) (Rankin et al., 2011), heat-shock inducible (Roose et al., 2009) and the Dex-inducible (Box 1) strategy (Zhuo et al., 2013). In addition, knocking down a specific protein can be titrated, either by using morpholinos at varying doses to reduce lethality (Eisen and Smith, 2008) or by microinjecting less mRNA of an inhibitor of the protein of interest (Baker et al., 1999). Alternatively, the CRISPR/Cas9 system can be utilized in *Xenopus* to bypass early developmental lethality caused by knocking out essential genes such as *lhx1* (DeLay et al., 2018). Human 17q12 deletions, which span two genes important for kidney development, *HNF1B* and *LHX1*, have been implicated in CAKUT (Nagamani et al., 2010). Although embryonically lethal in mice, *Xenopus* embryos survive *lhx1* knockout and demonstrate abnormal kidney development (DeLay et al., 2018). CRISPR/Cas9 editing results in mosaic knockout in *Xenopus*, which may explain why the knockout embryos were able to bypass embryonic lethality (DeLay et al., 2018), making this mosaic expression a silver lining. Furthermore, in *Xenopus tropicalis*, transplanting the primordial germ cells of CRISPR-mutagenized F_0_ embryos into a wild-type host, termed leapfrogging (Box 1), results in F_1_ embryos bypassing the embryonic lethality of the loss of the homeobox protein goosecoid (Blitz et al., 2016). Taken together, there are a range of techniques available to bypass embryonic lethality in *Xenopus*.

## Versatility in understanding cilia formation

Cilia are hair-like cellular protrusions that are found in most tissues in the body. Defects in genes important for ciliogenesis often lead to a vast array of phenotypes, including retinal degeneration, renal disease and congenital fibrocystic diseases of the liver (Waters and Beales, 2011). In addition to studying the ciliated A6 cell line derived from *Xenopus* and the primary cilia present *in vivo* in the pronephros, *Xenopus* embryos have a ciliated epidermis that has been useful for studying cilia function. *WDPCP* (Fritz) is one of 20 known genes that cause Bardet-Biedl syndrome (BBS; Box 1), all of which are involved in primary cilia function (Suspitsin and Imyanitov, 2016). Primary features of BBS include rod-cone dystrophy, genital abnormalities, learning difficulties, obesity and renal defects (Suspitsin and Imyanitov, 2016). The *Xenopus* epidermis has been used to study BBS because the cilia are easy to visualize. The authors found that Wdpcp localizes cytoskeletal proteins that control cell movement, called septins (Box 1), to the ciliary base (Kim et al., 2010a,b). Additionally, mutations in human *WDPCP* are linked to Meckel-Gruber (Box 1) syndrome (Kim et al., 2010a,b). This study demonstrates the versatility that the *Xenopus* model provides in understanding kidney diseases that extend beyond the pronephros.

## Future approaches

Until now, many studies utilized morpholinos to knock down protein expression. Additionally, the allotetraploid genome and long generation time (1 year) of *X.* *laevis* have previously made it less attractive for genetic experiments than the diploid *X.* *tropicalis* (Beck and Slack, 2001). However, the field is moving towards using CRISPR/Cas9 technology to generate gene knockouts. CRISPR/Cas9 gene editing in *Xenopus* is a rapid and inexpensive means of studying genes involved in developmental processes (Bhattacharya et al., 2015), introducing the possibility of performing large-scale genetic screens. Recently, the CRISPR/Cas9 system has been used in *Xenopus* to knock out an important kidney gene, *lhx1* (DeLay et al., 2018). Knock-in strategies using CRISPR/Cas9 have also been successfully utilized in *X.* *tropicalis* (Shi et al., 2015). Targeted CRISPR/Cas9 knock-in strategies facilitate the fusion of fluorescent tags to endogenous genes (Shi et al., 2015), allowing for more precise visualization of proteins *in vivo*. Furthermore, leapfrogging, which effectively circumvents a generation of breeding, allows researchers to generate transgenic lines quicker than with traditional breeding strategies (Blitz et al., 2016). These technologies are being favored over new criticisms concerning morpholino-driven phenotypes. Morpholinos have been recently shown to cause immune-response-related gene transcription and mistargeted splicing events. Although these effects cannot be completely ameliorated, researchers can take steps to reduce them, such as lowering the GC content and titrating the morpholino (Gentsch et al., 2018). Previous studies using morpholinos should not be discarded though, as our group's recent work suggests that they match CRISPR/Cas9 phenotypes (DeLay et al., 2019), but optimization to reduce unintended effects as well as proper controls should be utilized.

Distinctly, *Xenopus* has been used to study acute kidney injury and regeneration. Upon unilateral nephrectomy of the proximal tubules at stage 37/38 (∼53 hpf), *Xenopus* tadpoles are able to regenerate functional proximal tubules with no drug intervention (Caine and Mclaughlin, 2013; Suzuki et al., 2019). Additionally, *Xenopus* can even be used to generate kidney organoids that can replace the *Xenopus* kidney *in vivo* (Chan et al., 1999), which broadens the usefulness of this model organism for studying kidney disease. Because *Xenopus* oocytes are equipped with all the necessary machinery for development upon fertilization, they have long been used to study channel proteins. Given the large number of ion channels present in the kidney, this system can be readily exploited to study patient mutations in various channels and their effects on conductance. Additionally, two new transgenic frog lines have been developed in which GFP is expressed in the developing pronephros and mesonephros (Corkins et al., 2018), as well as in the pronephros alone (Ochi et al., 2012). This permits live imaging of the developing kidney, which can also be used to assess kidney disruption upon specific gene knockdown/out without the need for immunostaining or *in situ* hybridization. This new *Xenopus* line has the potential to streamline large-scale screens of kidney-disease-causing genes and of environmental factors that affect renal development. Also, bypassing the need to process embryos will expedite drug discovery screening in *Xenopus* (Schmitt et al., 2014), which may ultimately lead to clinical applications. The development of these new tools will undoubtedly advance our ability to study the genetic mechanisms underlying kidney development and disease.

## Limitations of *Xenopus* as a kidney model

Although *Xenopus* has many advantages that make it an attractive model for kidney development and disease, like all other models it possesses its own set of limitations. The most notable limitation may be that *Xenopus* is used to study the pronephros and has mesonephric nephrons that serve as its adult kidney instead of the metanephros of mammals. Although these differing nephrons maintain the same structure and function, this should be taken into consideration. Studying the pronephros may be different, but it offers a simplified method to study non-integrated individual nephrons. Many cases of CAKUT are associated with structural developmental defects such as vesicoureteral reflux, which cannot be visualized in *Xenopus* as they do not have a ureteric bud that forms a true collecting-duct system. Therefore, for malformations such as this, researchers should study *Xenopus* as a way to implicate new proteins in CAKUT and use in concurrence with another model system, such as mouse or kidney organoids, to study the anomalies more directly. Additionally, the *Xenopus* glomus does not have a Bowman's space, but instead blood filtrate goes directly into the coelomic cavity. Although this should be considered when conducting studies concerning the glomerulus, it may have a separate advantage. In mice, the glomerulus forms at the most proximal end of the S-shaped body, linking it to the development of the rest of the nephron. *Xenopus* however, can develop a glomus independently from the tubules, which may make understanding how the glomus forms more accessible (Urban et al., 2006). Lastly, *Xenopus* is an aquatic organism that does not require the resorption of water that is necessary in terrestrial animals.

## Conclusion

The studies reviewed here demonstrate that *Xenopus* is well suited to model congenital kidney diseases. *Xenopus* shares many genes and proteins with the mammalian kidney that have similar expression patterns and functions. As described here, many of the renal phenotypes observed in humans can be recapitulated in frogs. Next-generation DNA sequencing strategies, such as whole-exome sequencing, has helped identify candidate CAKUT-causing genes. An ideal organism to functionally assay these genes *in vivo* would develop quickly and produce large numbers of embryos, qualities that *Xenopus* possesses.

CAKUT accounts for 40-50% of pediatric chronic kidney disease worldwide (Vivante et al., 2014). Although important advances have been made in basic nephrology research, there are still missing pieces in the molecular basis of kidney development. This failure to fully grasp how the kidney develops translates into low cure rates for many of the genetic disorders that cause kidney disease. Being able to uncover novel genes and pathways that are involved in nephrogenesis will give us a better understanding of kidney development as a whole. This may lead to better treatment for patients with CAKUT, and its causative mutations may ultimately be eliminated with the application of gene therapy in human embryos. *Xenopus* is a very promising organism that can get us closer to accomplishing these goals.
